# A Mobile Health Intervention for Patients With Depressive Symptoms: Protocol for an Economic Evaluation Alongside Two Randomized Trials in Brazil and Peru

**DOI:** 10.2196/26164

**Published:** 2021-10-13

**Authors:** Daniela Vera Cruz dos Santos, Patrícia Coelho de Soárez, Victoria Cavero, Thaís I U Rocha, Suzana Aschar, Kate Louise Daley, Heloísa Garcia Claro, George Abud Scotton, Ivan Fernandes, Francisco Diez-Canseco, Lena Rebeca Brandt, Mauricio Toyama, Hellen Carolina Martins Castro, J Jaime Miranda, Ricardo Araya, Julieta Quayle, Paulo Rossi Menezes

**Affiliations:** 1 Departamento de Medicina Preventiva Faculdade de Medicina Universidade de São Paulo São Paulo Brazil; 2 CRONICAS Center of Excellence in Chronic Diseases Universidad Peruana Cayetano Heredia Lima Peru; 3 Faculdade de Enfermagem Universidade Estadual de Campinas Campinas Brazil; 4 CECS Centro de Engenharia, Modelagem e Ciências Sociais Aplicadas Universidade Federal do ABC São Bernardo do Campo Brazil; 5 Department of Medicine School of Medicine Universidad Peruana Cayetano Heredia Lima Peru; 6 Centre for Global Mental Health and Primary Care Research, Health Service and Population Research Institute of Psychiatry, Psychology, and Neuroscience King’s College London London United Kingdom

**Keywords:** cost-effectiveness, depression, diabetes, hypertension, noncommunicable diseases, randomized trials, low- and middle-income countries, mHealth, task shifting, behavioral activation

## Abstract

**Background:**

Mobile health interventions provide significant strategies for improving access to health services, offering a potential solution to reduce the mental health treatment gap. Economic evaluation of this intervention is needed to help inform local mental health policy and program development.

**Objective:**

This paper presents the protocol for an economic evaluation conducted alongside 2 randomized controlled trials (RCTs) to evaluate the cost-effectiveness of a psychological intervention delivered through a technological platform (CONEMO) to treat depressive symptoms in people with diabetes, hypertension, or both.

**Methods:**

The economic evaluation uses a within-trial analysis to evaluate the incremental costs and health outcomes of CONEMO plus enhanced usual care in comparison with enhanced usual care from public health care system and societal perspectives. Participants are patients of the public health care services for hypertension, diabetes, or both conditions in São Paulo, Brazil (n=880) and Lima, Peru (n=432). Clinical effectiveness will be measured by reduction in depressive symptoms and gains in health-related quality of life. We will conduct cost-effectiveness and cost-utility analyses, providing estimates of the cost per at least 50% reduction in 9-item Patient Health Questionnaire scores, and cost per quality-adjusted life year gained. The measurement of clinical effectiveness and resource use will take place over baseline, 3-month follow-up, and 6-month follow-up in the intervention and control groups. We will use a mixed costing methodology (ie, a combination of top–down and bottom–up approaches) considering 4 cost categories: intervention (CONEMO related) costs, health care costs, patient and family costs, and productivity costs. We will collect unit costs from the RCTs and national administrative databases. The multinational economic evaluations will be fully split analyses with a multicountry costing approach. We will calculate incremental cost-effectiveness ratios and present 95% CIs from nonparametric bootstrapping (1000 replicates). We will perform deterministic and probabilistic sensitivity analyses. Finally, we will present cost-effectiveness acceptability curves to compare a range of possible cost-effectiveness thresholds.

**Results:**

The economic evaluation project had its project charter in June 2018 and is expected to be completed in September 2021. The final results will be available in the second half of 2021.

**Conclusions:**

We expect to assess whether CONEMO plus enhanced usual care is a cost-effective strategy to improve depressive symptoms in this population compared with enhanced usual care. This study will contribute to the evidence base for health managers and policy makers in allocating additional resources for mental health initiatives. It also will provide a basis for further research on how this emerging technology and enhanced usual care can improve mental health and well-being in low- and middle-income countries.

**Trial Registration:**

ClinicalTrials.gov NCT12345678 (Brazil) and NCT03026426 (Peru); https://clinicaltrials.gov/ct2/show/NCT02846662 and https://clinicaltrials.gov/ct2/show/NCT03026426

**International Registered Report Identifier (IRRID):**

DERR1-10.2196/26164

## Introduction

Globally, depression affects more than 264 million people each year, with a high prevalence in Brazil and Peru (5.8% and 4.8%, respectively). In Brazil, depressive disorders are ranked as the fourth leading cause of years lived with disability, and thirteenth for causing disability-adjusted life years [1]. In Peru, depressive disorders account for 37% of the years lived with disability attributed to mental health conditions and are responsible for almost 46,000 disability-adjusted life years [2].

Chronic diseases such as hypertension and diabetes can be exacerbated by depression, which is often undiagnosed and thus remains untreated [3,4]. This can lead reduced quality of life, increased physical health complications, and eventually a rise in treatment costs for both the individual and the health economy [4].

In low- and middle-income countries (LMICs) access to mental health care remains extremely limited and unequal, with those living in low-resource areas facing greater difficulties in accessing mental health care [5,6]. This is due to limited resource availability and a lack of trained health personnel. For instance, in Brazil there are only 4.48 specialists per 100,000 inhabitants. A similar scenario is observed in Peru, with 2.06 psychiatrists and 6 psychiatric nurses per 100,000 people [7]. Most Brazilians and Peruvians with clinically relevant levels of depressive symptoms currently do not receive any treatment. There is thus a clear need to address the mental health treatment gap within these settings.

Mobile health (mHealth) interventions offer potentially scalable and affordable solutions to reduce the treatment gap in mental health care (eg, depression). Studies on the effectiveness of mHealth intervention in this regard have shown promising results [8-11].

The Latin America Treatment and Innovation Network in Mental Health (LATIN-MH), 1 of 5 hubs funded by the National Institute of Mental Health (NIMH), conducted 2 randomized controlled trials (RCTs) evaluating the effectiveness of a low-intensity behavioral activation intervention for depression (CONEMO). These aimed to reduce symptoms of depression among individuals with hypertension, diabetes, or both conditions attending public health care facilities in São Paulo, Brazil, and Lima, Peru [12].

The acronym CONEMO is derived from the terms “Control” and “Emotional” (meaning: emotional control). The intervention is delivered via a smartphone app, and minimally supported by a nurse/nurse assistant (NA). The app consists of 18 brief mini-sessions, delivered automatically over a 6-week period, with 3 mini-sessions per week. Each session requires less than 10 minutes to complete [12]. The CONEMO intervention seeks to expand access to mental health care while using the available health services in Brazil and Peru [13].

Although efforts have been made to ensure the effectiveness, feasibility, and affordability of similar interventions, the success and impact are primarily determined by regional differences in cost structures and the burden of chronic diseases [14]. Cost data provide essential information for financial planning and enable decisions to be made regarding implementation feasibility. This cost analysis may provide a critical contribution to the planning and prioritization of interventions to address the large and growing burden of depression in LMICs.

Although there is an increase in economic evaluations of mHealth interventions, evaluations of these interventions in LMICs remain limited [15]. This paper describes a protocol for an economic evaluation to be conducted as part of 2 RCTs evaluating the cost-effectiveness of CONEMO, an mHealth intervention to treat symptoms of depression in people with diabetes or hypertension or both in Brazil and Peru.

## Methods

### Study Design

The economic evaluation will follow the Methodological Guidelines for Economic Evaluation Studies of Health Technologies [16] and will be reported according to the Consolidated Health Economic Evaluation Reporting Standards (CHEERS) checklist [17]. The multinational economic evaluations will be fully split analyses (incorporating estimates of both resource use and clinical effectiveness from the same group of patients from individual countries) with a multicountry costing approach (applying unit cost estimates from individual countries to account for resource use in each of those countries) [18]. The within-trial analyses will be conducted using data from the randomized control trials NCT02846662 and NCT03026426, and secondary data on health service costs extracted from governmental public databases. This protocol outlines the methods for a cost-effectiveness analysis and cost-utility analysis alongside the RCTs conducted in Brazil and Peru, respectively.

The proposed economic evaluations will be conducted from both the public health care system perspective (including only health care system costs) and the societal perspective (including health care system costs plus patient and productivity costs).

### Study Population

All participants in the RCT will be included in the economic evaluations. Participants include patients of the public health care system in Brazil or Peru. Inclusion criteria were adults (age ≥21 years), able to read the screen of a smartphone, receiving treatment for hypertension, diabetes, or both (self-report validated with clinical records), and experiencing symptoms of depression. Symptoms of depression were defined as obtaining a score of 10 or more on the 9-item Patient Health Questionnaire (PHQ-9) [19]. Participants were recruited from 20 primary care units in São Paulo, Brazil, and from 4 primary care centers and 3 outpatient clinics in Lima, Peru.

### Setting and Location

The Brazilian health care system is made up of 2 subsectors: the National Health System (Sistema Único de Saúde [SUS]), which is universal and free for everyone, and private health insurance, which covers approximately 25% of the population. Peru has a decentralized health care system administered by 5 providers: the Ministry of Health (MINSA), which provides health services for 60% of the population; Social Security (EsSalud), which covers 30% of the population; and the Armed Forces (Fuerzas Armadas Españolas [FFAA]), Policía Nacional del Perú (PNP), and private sector, which together provide services to the remaining 10% of the population.

Both Brazilian and Peruvian health care systems have been implementing several reforms over the last 2 decades. As a result, health service networks in these countries are segmented into primary, secondary, and tertiary care levels [6,20]. Patients with diabetes and hypertension are typically seen within primary care in Brazil (primary care units) and within either primary (primary care centers) or secondary care units (hospital outpatient clinics) in Peru.

The trials have been conducted in primary care settings (20 public primary care centers of the SUS) in Sao Paulo, Brazil, and in 4 primary care centers in EsSalud and 3 outpatient clinics in MINSA in Lima, Peru [21].

### Comparators

In the Brazilian and Peruvian health care systems, there is no screening policy for mental health in primary care units, where the focus is on physical health. Mental health care is available within specialized primary and secondary care services in both countries. The usual care in the primary care setting integrates practices of health promotion and clinical practice, which do not include the use of depression screening instruments.

The economic evaluations will compare CONEMO plus enhanced usual care with only enhanced usual care.

### CONEMO Plus Enhanced Usual Care

The intervention group received CONEMO plus enhanced usual care. CONEMO is a low-intensity mHealth intervention aimed at reducing symptoms of depression. It is delivered via a smartphone app, and is minimally supported by a nurse/NA. The CONEMO app delivers 18 brief, automated mini-sessions over a 6-week period, with 3 mini-sessions per week. Each session is completed in less than 10 minutes.

The app content is based on behavioral activation, an evidence-based psychological approach to treat depression. Processed data on app usage were collected automatically and reviewed in real-time by nurses/NAs through a dashboard installed on tablets.

Nurses/NAs met with participants for an initial face-to-face meeting, where participants received a smartphone with the app preinstalled and completed a tutorial on its use. Nurses/NAs made 2 mandatory phone calls to all CONEMO participants at the beginning of the program to troubleshoot any difficulties and enhance motivation for the use of CONEMO. Additional calls were prompted through notifications sent to nurses/NAs when the CONEMO automated system detected nonadherence. Patients could request technical help through the help button on the app. Nurses/NAs received training and were supervised weekly by psychologists through face-to-face meetings or via telephone.

### Enhanced Usual Care

The control group received the usual care plus the following procedures:

- Participants were screened using the PHQ-9 at baseline and at 3- and 6-month follow-ups.
- Those with a score of 10 or more on the PHQ-9 were advised to seek appropriate mental health care.
- Those presenting with suicide risk were referred to specialized mental health services, by either a health professional (in Sao Paulo and Lima) or the research team (in Lima). In Lima, relatives or health care professionals were also informed depending on risk severity.
- All participants received at least one call after screening at inclusion, and at 3- and 6-month follow-up from the research team to assess if they had sought specialized mental health care. Those with higher risk or who did not seek care received additional phone calls.

### Time Horizon and Discount Rate

Time horizon analyses will use the study follow-up period: 3 and 6 months after the inclusion of participants in the study in order to understand its permanence effect. We truncated time horizons to the length of follow-up in the RCTs because the long-term health outcomes remain unclear and are not expected to last beyond 6 months.

Following the recommendations of the Methodological Guidelines for Economic Evaluation Studies of Health Technologies [16], we will not apply the standard discount rate of 5% on costs and benefits, because of the short (<1 year) time horizons.

### Choice of Health Outcomes and Measurement of Effectiveness

Table 1 summarizes the health outcome measures and time of collection for the outcomes that will be used in the economic evaluations.

Two health outcomes will be measured: reduction in symptoms of depression and health-related quality of life. First, the primary outcome of effectiveness for the cost-effectiveness analysis will be the proportion of patients with at least 50% reduction in PHQ-9 scores. This indicator has been used in many depression trials and is considered a robust way of symptom improvement [22].

Second, the EuroQol 5-dimensional questionnaire, 3-level version (EQ-5D-3L) will be used to measure health-related quality of life and provide utilities for estimation of quality-adjusted life years (QALYs). The EQ-5D-3L is a generic preference-based measure used in many clinical trials for a wide range of health conditions and treatments [23].

The measurement of clinical effectiveness will take place over 3 periods: baseline, 3-month follow-up, and 6-month follow-up. Both countries used the same measures (PHQ-9 and EQ-5D-3L).

**Table 1:** Summary of health outcomes measured.

Health outcomes	Measures	Timing of collection	Source of data
Depressive symptoms	PHQ-9^a^	Baseline, 3-month follow-up, and 6-month follow-up	Trials’ patient-reported outcomes
Quality of life	EQ-5D-3L^b^	Baseline, 3-month follow-up, and 6-month follow-up	Trials’ patient-reported outcomes

^a^PHQ-9: 9-item Patient Health Questionnaire.

^b^EQ-5D-3L: EuroQol 5-dimensional questionnaire, 3-level version.

### Estimating Resources and Costs

The cost estimation will involve 3 stages: (1) identification of the costing items used to provide a particular service, (2) measurement of the resources used, (3) application of monetary value for each cost item, and calculation of the unit cost of a particular service [24].

The measurement of the resources will take place over 3 periods, baseline, 3-month follow-up, and 6-month follow-up, in the intervention and control groups. These are the same periods used to assess clinical effectiveness in the RCTs.

The measurement of resource use will be based on the group of patients from each country. The costing approach will apply unit cost estimates from Brazil and Peru to account for resource use in each of those countries.

Costs will be applied to the resources based on the unit costs or price weights most recently published in the respective official government databases (Department of Informatics of the National Health System, Departamento de Informática do Sistema Único de Saúde [DATASUS] in Brazil; and the Integral Health Insurance/Seguro Integral de Salud [SIS] in Peru). Other resource use not recorded in DATASUS or SIS will be based on LATIN-MH databases. These costs will be obtained in Brazilian reals (R$) and Peruvian sols (S$), as per rates in 2017. All costs will be adjusted for inflation using the Brazilian and Peruvian consumer price indexes for the current reference year (2021) and will be converted into international dollars (INT$) using the 2021 purchasing power parity (PPP) conversion factors for Brazil and Peru.

We will use a mixed costing methodology (ie, a combination of top–down and bottom–up approaches) considering 4 cost categories: intervention (CONEMO related) costs, health care costs, patient and family costs, and productivity costs. Table 2 presents an overview of resource use and cost measures that will be used in the economic evaluations. These include costs hypothesized to differ between CONEMO plus enhanced usual care (the intervention group) and enhanced usual care (the control group).

**Table 2:** Summary of cost categories measured.

Cost category and unit					Unit cost		Source of data	
**Intervention (CONEMO^a^) costs**								
	Staff involved in supervision		Hour of supervision	Cost/hour		LATIN-MH^a^ databases		
	Staff involved in training		Hour of a research assistant	Cost/hour		LATIN-MH databases		
	Transport		Number of trips	Cost/trip		LATIN-MH databases		
	Dashboard and app maintenance		Maintenance charge	Cost/month		LATIN-MH databases		
	Internet package		Monthly package	Cost/month		LATIN-MH databases		
	Office supplies			Cost/month		LATIN-MH databases		
	Infrastructure			Cost/month		LATIN-MH databases		
**Health care costs**								
	Psychiatrist visits		Number of visits	Cost/visit		CEI^b^ and ISSM^c^		
	Family physician visits		Number of visits	Cost/visit		CEI and ISSM		
	Psychologist visits		Number of visits	Cost/visit		CEI and ISSM		
	Emergency room visits		Number of visits	Cost/visit		CEI, ISSM, and SAEs^d^		
	Hospital admissions		Number of hospital admissions	Cost/hospital admission		CEI, ISSM, and SAEs		
	Medicines		Number of doses	Cost/dose		CEI and ISSM		
**Patient and family costs**								
		Transport	Number of trips	Cost/trip		Average cost for public transport		
		Internet	Monthly package	Cost/month		Average market price		
**Productivity costs**								
		Days of work lost	Number of days	Cost/hour		Average hourly wage		

^a^CONEMO: control emotional (emotional control intervention).

^b^LATIN-MH: Latin America Treatment and Innovation Network in Mental Health.

^c^CEI: Cost-Effectiveness Instrument.

^d^Mental Health Instrument.

^e^SAE: Serious Adverse Events Report.

### Intervention Costs

Intervention costs include the value of the staff time and transport involved in either supervision or training of the primary care workers that will use CONEMO.

We will assume that the intervention (CONEMO) is operational, and so start-up costs, such as design and development of CONEMO, will be excluded. Therefore, we will include only costs for maintenance of the dashboard and app, internet package costs, and expenditures related to office supplies and infrastructure.

All of the research-related costs associated with trial administration, data collection, and outcome assessment of RCTs will be excluded.

### Health Care Costs

Health care costs will include direct medical costs: outpatient visits, emergency room and hospital admissions, and medicines prescribed during the study period.

### Patient and Family Costs

Patient and family costs will include direct nonmedical costs, such as transport for seeking health care and internet expenditures. In São Paulo, we will assume that for each hospital admission, there will be 2 public transport trips. For those aged over 60, we will add a companion cost (according to Article 16 of the Brazilian Elderly Statute) [25]. In case of attendance at the primary care centers, no transportation cost will be calculated, as we are assuming participants will be attending a local service. In Lima, we will consider the use of 2 public transport trips for all participants. For participants aged over 60, considering the lack of specific regulations, we agreed on including an additional 50% cost for caregiver transportation.

We assumed that the patients already own a smartphone and included only the costs of internet access.

### Productivity Costs

Productivity costs will include indirect costs related to lost days of work due to morbidity and will be estimated according to the human capital approach [26]. Productivity costs associated with depressive symptoms will be calculated by multiplying the number of hours absent with an average hourly wage in Brazil and Peru.

### Policy Statement, Data Analysis, and Security Responsibilities

The RCTs were approved by the Data and Safety Monitoring Board (DSMB) of the NIMH, USA, and local ethics committees in São Paulo and Lima.

Primary data (phase 1) were collected on ethical standards and underwent internal and external audit by the DSMB. Secondary data (phase 2) will follow the same guidelines for quality purposes.

Informed consent was obtained from participants by the research team at screening and baseline. Consent for this study was obtained as part of the RCT procedures.

Study coordinators (DdS and PS) will carry out the collection and analysis of secondary data from each country with the support of the LATIN-MH Data Center team and a specialized consultant under the supervision of the principal investigators (PM and RA). Data sets will be anonymized by removing personal identifiers (eg, name, phone number) to protect the confidentiality of study participants.

### Analytic Methods

Mean values of the estimated health outcomes and costs, as well as mean differences between the comparator groups (CONEMO plus enhanced usual care versus enhanced usual care) will be reported. Incremental cost-effectiveness ratios (ICERs) will be calculated as the arithmetic mean difference in cost between CONEMO plus enhanced usual care and enhanced usual care, divided by the arithmetic mean difference in effect. For the cost-effectiveness analysis, the corresponding ICER will be expressed as the incremental costs per at least 50% reduction of PHQ-9 scores. In the cost-utility analysis, the ICER will be expressed as the incremental costs per QALY gained. ICERs will be generated by nonparametric bootstrapping methods using random resamples for every 1000 pairs of outcomes and costs for the intervention and comparator groups to derive 95% CIs [27]. The distribution of mean incremental costs and outcomes shown on cost-effectiveness will be conducted to test the robustness of results.

### Sensitivity Analyses

Deterministic and probabilistic sensitivity analyses will be performed to determine the level of confidence around the resulting ICERs. Several deterministic (1 way) sensitivity analyses will be carried out by varying the key parameters (such as quantity of resource use, unit cost of resource use, QALYs) in the within-trial analyses. Results from deterministic sensitivity analyses will be presented in a tornado diagram to compare the relative importance of the parameters with the ICER. The results from the probabilistic sensitivity analysis will also be presented using cost-effectiveness acceptability curves [28]. This curve indicates the probability that the CONEMO plus enhanced usual care is cost-effective compared with enhanced usual care, considering the thresholds range of $PPP3210-$PPP10,122 per QALY gained for Brazil, and of $PPP1969-$PPP7747 per QALY gained for Peru [29].

### Statistical Software Use for Health Economic Analysis

Stata (StataCorp) version 15.0 or higher will be used for all health economics analyses.

## Results

Phase 1 of the project ran from September 2016 to April 2018 to assess the applicability of CONEMO. Phase 2 began in June 2018 and is expected to be completed in September 2021. The economic evaluation of CONEMO will be conducted during this phase. Results will be used to report the costs of CONEMO to help plan the implementation of the intervention. The evaluation will also calculate the cost-effectiveness of CONEMO to improve symptoms of depression.

## Discussion

### Overview

Assessing the economic viability of the CONEMO intervention based on its cost-effectiveness will enable us to ascertain the feasibility of its implementation in primary and secondary care. If shown to be financially viable, the intervention could help bridge the treatment gap and increase access to mental health care. If the ICER is favorable, there would be a strong case for policymakers to support the scale-up of this intervention.

Implementing the CONEMO intervention would enable the screening, identification, and treatment of depression in patients with chronic diseases. The expected result is a reduction in symptoms and likely an increase in self-care, adherence to treatment, and quality of life. These outcomes could also result in fewer complications and required resources, as well as reduced health care costs. Using nursing staff to support patients rather than specialized mental health professionals and specialized services could also be an efficient use of human resources, thereby saving costs in the longer term.

Economic evaluations are particularly important because they can be used to guide decision makers or funders in determining if the mHealth interventions improve health outcomes when compared to other existing interventions [15]. They are also important for analyzing whether the cost to adopt and maintain the intervention in a health system is justified. One of the barriers of mHealth implementation and scale-up is the financial feasibility, yet few studies include or detail operational costs of mobile communication, such as fees charged to end users (patients).

### Generalizability and Future Research

If found to be clinically effective and cost-effective, CONEMO could be used in other populations with similar treatment gaps [13]. Replication has been made possible by the standardized materials used. The generalizability of results is likely high due to the diverse in-country heterogeneous sample and its implementation in 2 countries.

- Implications for health provision and use: We would seek to collaborate with other services to develop further evidence for implementation.
- Implications for health policies: Despite some limitations, this protocol will be used to measure the costs of mental health services in 2 studies, generating data to support local mental health policies, boost research in the country, and support new studies.

### Study Limitations

Health economic evaluation studies on mHealth interventions have not been conducted in Brazil and Peru; while this is an innovative research study, some limitations remain. These are as follows:

- Cost-effectiveness analysis may be hampered by the scarcity of economic and epidemiological data available and the composition of the variables for each cost when found.
- Peruvian analysis will follow Brazilian guidelines due to the lack of guidelines for health economic evaluation in Peru.
- Some cost items will be based on other similar items, which do not express the same precision level of a specialized service sample collected on official cost indicator databases.
- Difficulty in including all indirect costs incurred from the societal perspective due to insufficient information related to the treatments under analysis.

## Abbreviations

CONEMO: control emotional (emotional control intervention)
DATASUS: Departamento de Informática do Sistema Único de Saúde (Department of Informatics of the National Health System)
EQ-5D-3L: EuroQol 5-dimensional questionnaire, 3-level version
FFAA: Fuerzas Armadas
ICER: incremental cost-effectiveness ratio
LATIN-MH: Latin America Treatment and Innovation Network in Mental Health
LMIC: low- and middle-income countries
mHealth: mobile health
NA: nurse assistant
PHQ-9: 9-item Patient Health Questionnaire
PPP: purchasing power parity
QALY: quality-adjusted life year
RCT: randomized controlled trial
SIS: Seguro Integral de Salud (Integral Health Insurance)
